# Enhancing *Trichoderma* efficacy in managing wheat stem rust disease and boosting production through the application of certain chemical inducers

**DOI:** 10.1186/s12870-025-06434-9

**Published:** 2025-04-30

**Authors:** Hany H. A. El-Sharkawy, Nada A. Mostafa, Safaa A. M. Yousef, Salama A. S. El-Blasy, Osama Abd El Badeea

**Affiliations:** 1 Mycology and Diseases Survey Research Department, Plant Pathology Research Institute, Agricultural Research Center, Giza, Egypt; 2 Agricultural Botany Department, Faculty of Agriculture, Mansoura University, Mansoura, Egypt; 3 Mycology and diseases Survey Research Department, Plant Pathology Research Institute, Agricultural Research Center, Giza, Egypt; 4 Leguminous and Forage Crop Diseases Department, Plant Pathology Research Institute, Agricultural Research Center, Giza, Egypt; 5 Wheat Diseases Research Department, Plant Pathology Research Institute, Agricultural Research Center, Giza, Egypt

**Keywords:** Wheat, Stem rust, Induced resistance, Electron microscope, *T. harzianum*

## Abstract

**Background:**

Wheat stem rust (WSR), caused by *Puccinia graminis* f. sp. *tritici* (Pgt), represents a significant threat to global wheat production. Biocontrol agents, such as *Trichoderma harzianum* HE22 (TH), offer sustainable strategies for managing this disease. This study evaluates the efficacy of TH cultured in potato dextrose broth (PDB) supplemented with various chemical inducers (TSDCIS), including potassium tartrate (T1), a mixture of micronutrients (T2), and thiamine (T3). These treatments were compared to unmodified TH (T4) to evaluate their potential in controlling WSR at both the seedling stage and under field conditions, with the primary objective of enhancing disease management while improving wheat yield and quality.

**Results:**

Under both greenhouse and field conditions, T1 significantly reduced disease severity by 86.2% and 77.7%, respectively, and decreased the area under the disease progress curve (AUDPC) by 77.5% compared to the untreated control. T1 also extended the incubation and latent periods, reduced pustule density, and mitigated oxidative damage. Biochemical analyses revealed elevated levels of total phenols and enhanced activity of antioxidant enzymes, including peroxidase (POD) and polyphenol oxidase (PPO), along with increased concentrations of ascorbic acid and proline. Additionally, T1 reduced lipid peroxidation, lowered H₂O₂ concentrations, and minimized electrolyte leakage, demonstrating its protective effects on plant tissues. Microscopic analyses using transmission electron microscopy (TEM) and scanning electron microscopy (SEM) confirmed these protective effects. Treated plants exhibited intact cellular membranes, well-organized chloroplasts, and enhanced cellular integrity, whereas untreated plants showed severe structural damage, including plasmolysis and distorted chloroplast morphology.

**Conclusion:**

*T. harzianum* HE22 supplemented with potassium tartrate (T1) demonstrates significant potential as an environmentally friendly and highly effective strategy for managing wheat stem rust. This approach not only reduces disease severity but also enhances wheat yield and overall plant health, making it a promising tool for sustainable agricultural practices.

## Background

Wheat (*Triticum aestivum* L.) is the world’s most important cereal crop, with a global production of approximately 7.97 billion tons cultivated across 2.2 billion hectares in 2020 [1]. However, wheat production faces significant threats from various pathogens that negatively impact both grain quality and yield. Among these, WSR, caused by Pgt, is one of the most devastating diseases. Under favorable conditions, WSR can decimate entire wheat fields within a month, resulting in the loss of millions of hectares [2]. Although chemical fungicides are widely used and effective in controlling WSR, their application poses potential health risks to humans, animals, non-target plants, and biodiversity [3]. This highlights the urgent need for safer, more sustainable alternatives to chemical fungicides for managing wheat stem rust.

*T. harzianum* exerts its biocontrol effects both indirectly, by inducing plant resistance, and directly, through completion for nutrients and space, secretion of extracellular hydrolytic enzymes, and production of both volatile and non-volatile toxic metabolites Additionally, *T. harzianum* releases antagonistic compounds that specifically target fungal pathogens [4–6]. These multifaceted attributes make *Trichoderma* species valuable components of integrated pest management programs.

Beyond disease control, *T. harzianum* promotes plant growth by activating phytohormones such as indole-3-acetic acid, gibberellins, and harzianic acid, which enhance yield, enhance yield, metabolism, cell division, and chlorophyll synthesis—contributing to overall plant health and productivity [2]. El-Sharkawy et al. [2] demonstrated that combining *Trichoderma* species with arbuscular mycorrhizal fungi effectively suppresses wheat stem rust by inhibiting pathogen spore germination and enhancing plant defense responses.

Despite its potential, the effectiveness of *T. harzianum* in controlling WSR can be influenced by variations in environmental conditions, soil types, and pathogen pressures, which can impact its biocontrol efficacy. To enhance its performance, chemical additives such as potassium tartrate, thiamine, and micronutrient mixtures have been investigated. These additives improve spore production, fungal growth, and the synthesis of antifungal metabolites, including hydrocarbons, alcohols, ketones, and terpenes, which suppress pathogens like *Rhizoctonia solani* and activate plant defense mechanisms, boosting plant resistance to disease. Yousef et al. [7] reported that *T. harzianum* produces these compounds when cultured with such stimulants. Additionally, studies by Merid and Zafari [8] demonstrated that manganese enhances chitinase activity in *T. brevicompactum* and *T. koningiopsis*, improving pathogen control. Antioxidants promote fungal growth, stimulate resistance structure formation, and enhance secondary metabolite production. Therefore, supplementing *T. harzianum* with these stimulants enhances its biocontrol capabilities, making it a more effective agent against WSR and other fungal pathogens.

This synergistic enhancement of metabolite production positions *T. harzianum* as a highly effective and environmentally friendly biocontrol agent capable of managing plant diseases while promoting plant health in agricultural systems. This study aimed to optimize the biocontrol potential of *T. harzianum* against WSR by incorporating specific chemical additives into its growth medium under both greenhouse and field conditions. Additionally, the study seeks to evaluate the impact of these treatments on wheat yield, as well as on ultrastructural and biochemical responses.

## Methods

### Fungal inocula, *Trichoderma*, chemical materials, and cultivar

Fresh urediniospores of *Puccinia graminis* f. sp. *tritici* inoculum were kindly provided by the Wheat Diseases Research Department, while *Trichoderma harzianum* HE22 was kindly supplied by the Mycology Research and Diseases Survey Department, both of which are affiliated with the Plant Pathology Research Institute, Agricultural Research Center (ARC), Egypt. Five-millimeter mycelial discs from a seven-day-old culture of *T. harzianum* were inoculated into 500 mL flasks containing 100 mL of potato dextrose broth (PDB) supplemented with various tested materials, including potassium tartrate (30 mg), a micronutrient mixture (Zn sulfate, 50 mg; Mn sulfate, 50 mg; boric acid, 20 mg; selenium, 0.01 mg), and thiamine (1 mg). The final concentration of *T. harzianum* was adjusted to 10⁸ cfu mL⁻1 before application.

The seeds of the susceptible Morocco wheat genotype were generously provided by ARC. Crown fungicide (El-Helb Pesticides Co., Egypt), containing propiconazole as its active ingredient, was applied at a concentration of 0.3 mL L⁻1. All other chemicals were procured from Al-Gomhouria Co., Egypt. A suspension of urediniospores was prepared at a concentration of 104 spores mL⁻1. The experiments were conducted at both the seedling and field stages.

### Seedling stage experiment

Ten Morocco wheat seeds were sown in seven-centimeter plastic pots filled with clay soil, irrigated, and fertilized regularly. Each treatment was replicated in three pots. The inoculation protocol followed the method described by Stakman et al. [16]. Seven-day-old seedlings were sprayed with the following treatments:

- **T1**: *T. harzianum* supplemented with potassium tartrate.
- **T2**: *T. harzianum* supplemented with micronutrients. 
- **T3**: *T. harzianum* supplemented with thiamine.
- **T4**: *T. harzianum* without supplements.
- **Control (fungicide)**: Crown fungicide (0.3 mL L⁻1).
- **Untreated control**: Sprayed with water only.

Seventy-two hours after treatment application, seedlings were inoculated with uredospore suspension. The pots were covered with transparent plastic hoods for 48 h at 20–25 °C and 90% relative humidity to facilitate infection. They were then transferred to a greenhouse maintained at 20–24 °C (day/night), with a 16-h of photoperiod, and 60% relative humidity [9].

### Disease assessment

#### Disease assessment

Disease severity (DS) was assessed following the diagrammatic scale (1–100%) proposed by Peterson et al. [10]. The infection type (IT) was classified into seven categories as described by Stakman et al. [11], ranging from immune to highly susceptible. The average coefficient of infection (ACI) was determined by multiplying disease severity by the constant values assigned to infection types (R = 0.2, MR = 0.4, MS = 0.8, S = 1), as per Johnston and Browder [12].

#### Biochemical analysis

The activities of defense-related enzymes in wheat leaves were analyzed using established protocols:

- Polyphenol oxidase (PPO) activity was measured following the method of Maria et al. [13].
- Peroxidase (POD) activity was determined according to Maxwell and Bateman [14].
- Total phenolic content was quantified using the Folin-Ciocalteu reagent, following Malik and Singh [15].
- Hydrogen peroxide (H₂O₂) levels were estimated using the method of Jana and Choudhuri [16].
- Proline content was calculated following Arbona et al. [17].
- Ascorbic acid levels were measured using the method of Law et al. [18].
- Lipid peroxidation, expressed as malondialdehyde (MDA) content, was determined following the protocol of HongBo et al. [19].
- Photosynthetic pigment levels (chlorophyll and carotenoids) were analyzed using Harborne’s method [20].

Leaf samples were collected two weeks after pathogen inoculation, and all biochemical assays were performed with four replicates per treatment for statistical accuracy.

#### Electron microscopic studies

Seven days post-inoculation, wheat leaves were sampled for scanning electron microscopy (SEM) analysis. Leaf Sects. (1 cm2) underwent a dehydration process using a graded ethanol series (10% to 100%) with 10-min intervals at each concentration. The dehydrated samples were dried using a critical point dryer (TEC–030) and subsequently coated with gold using a sputter coater (FDU-010). SEM images were captured using a JEOL 100 CX-II ASID-4D microscope (Tokyo, Japan) to determine the average number of open and closed stomata within a field area of 0.195 mm2 at 250 × magnification.

Fourteen days post-inoculation, wheat leaf samples were processed for transmission electron microscopy (TEM). Leaf Sects. (1 mm2) were fixed in 2.5% glutaraldehyde in 0.1 M sodium cacodylate buffer (pH 7.0) for 24 h at 22 °C. Samples underwent ethanol dehydration (10% to 100%) in 10-min increments and were embedded in a mixture of ethanol and propylene oxide before transfer to gelatin capsules containing fresh Araldite. The samples were polymerized at 60 °C for 60 h.

Ultrathin sections were prepared using a Reichert Ultramicrotome with a glass knife, mounted on 200-mesh copper grids, and stained with uranyl acetate and lead citrate. TEM observations were performed using a JEM-1230 transmission electron microscope (JEOL Ltd., Tokyo, Japan) to assess ultrastructural changes in mesophyll cells.

#### Field experiments

Field trials were conducted at the experimental farm of El-Noubaria Agricultural Station, El-Behera Governorate, Egypt (latitude 30.8661° N, longitude 30.2625° E) during two consecutive growing seasons: December 15–20 to May 1–5 in 2020/2021 and 2021/2022. The field was plowed, laser-leveled, and divided into six plots (3 m^2^ each), with three rows per plot (25 cm apart). Wheat seeds were sown at a rate of 5 g per row.

Fertilization included superphosphate (0.06 units) and nitrogen (0.32 units). Environmental conditions—average temperature (15–28 °C), relative humidity (60–80%), and soil type (sandy loam, pH 7.8)—were monitored for uniformity. A completely randomized block design (CRBD) with three replicates per treatment was used. Pathogen inoculation was performed at the booting stage (92 days after planting), using 1 g of *P. graminis* f. sp. *tritici* urediniospores mixed with 20 g of talcum powder, applied to spreader plants in the evening to maximize humidity for germination.

Biocontrol treatments involved *T. harzianum* cultured in PDB with chemical inducers, adjusted to 10⁸ CFU mL⁻^1^. Treatments were applied three days prior to inoculation as follows:

- T1: *T. harzianum* supplemented with potassium tartrate
- T2: *T. harzianum* supplemented with micronutrients 
- T3: *T. harzianum* supplemented with thiamine
- T4: *T. harzianum* (without supplements)
- Control (fungicide): Crown fungicide (0.3 mL L⁻.^1^)
- Untreated control: Water spray only

Each treatment was replicated three times under CRBD for statistical robustness.

#### Disease assessments

Disease severity (DS) was assessed using a diagrammatic scale ranging from 1 to 100%, as described by Peterson et al. [10]. The area under the disease progress curve (AUDPC) was calculated according to the method outlined by Pandey et al. [21], offering a comprehensive evaluation of disease progression over time.

#### Yield assessment

At maturity, yield components were analyzed to evaluate the effectiveness of the treatments. Measurements included plot grain yield and the weight of 1000 kernels (g). To ensure consistency, all weights were standardized by drying samples in a hot air oven at 80 °C to achieve a uniform moisture level.

#### Statistical analysis

Data were analyzed using CoStat software (CoHort Software, USA, version 6.4) [22]. Treatment means were compared using Duncan’s multiple range test at p ≤ 0.05 [23].

## Results

### Influence of TSDCIS on stem rust disease reduction in wheat (Morocco Genotype) at the seedling stage under greenhouse conditions

**Fig. 1:**
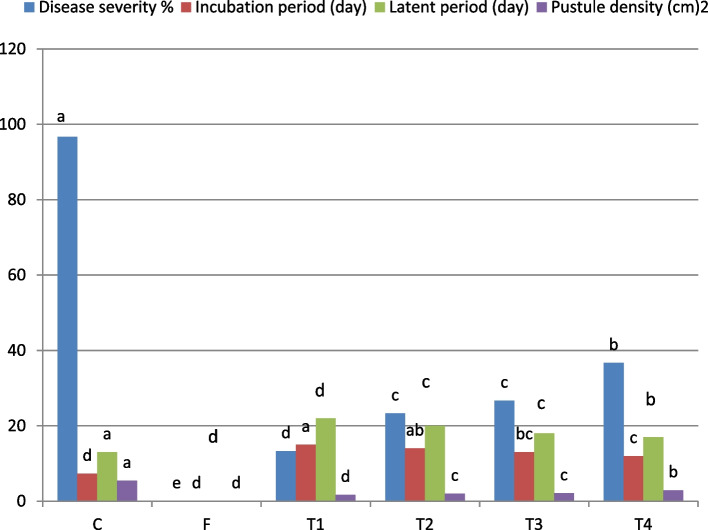
Effect of TSDCIS on the disease parameters of stem rust Morocco genotype at seedling stage under greenhouse

The data presented in Fig. 1 highlight the significant impact of TSDCIS treatments on reducing stem rust disease severity (DS) in the Morocco wheat genotype under greenhouse conditions. Among the treatments, T1 exhibited the highest efficacy, resulting in the smallest pustules, reduced pustule density, and the formation of distinct yellow halos—indicative of enhanced plant resistance.

In contrast, **T4** exhibited the least disease suppression, with larger and denser pustules compared to the other treatments.

This treatment also recorded the longest incubation (15 days) and latent periods (22 days), effectively delaying symptom onset and suppressing pathogen development. T2 provided moderate reductions in DS and pustule density, improving plant health compared to untreated controls, while T3 delayed symptom appearance and minimized tissue damage by activating systemic resistance pathways. In contrast, T4 provided limited disease suppression, resulting in larger and denser pustules relative to the other treatments.

### Impact on antioxidants enzymes and total phenols

Table 1 summarizes the effects of TSDCIS treatments on biochemical defense parameters in wheat plants (Morocco genotype) infected with *P. graminis* f. sp. *tritici* under greenhouse conditions. The treatments significantly enhanced total phenolic content, antioxidant enzyme activities (POD and PPO), proline, and ascorbic acid levels compared to the control and fungicide treatments. Among the treatments, T1 recorded the highest enzyme activities (POD: 74.8 U/g F.W; PPO: 17.9 U/g F.W) and phenolic content (1866.3 mg/g F.W), while T4 exhibited the lowest improvements among TSDCIS treatments. The control and fungicide treatments recorded the lowest values across all parameters.

**Table 1:** Effect of TSDCIS on phenolic content, the activity of the antioxidant enzymes polyphenol oxidase, peroxidase, proline, ascorbic acid in the leaves of wheat plants (Morocco genotype) infected with *Puccinia graminis* f. sp. *tritici* three days after infection at the seedling stage under greenhouse conditions

Treatment	POD activity (Unit/g F. W)	PPO activity (Unit/g F. W)	Total phenols (mg/ g F. W)	Proline µg/g F. W	Ascorbic acid (mg/g F. W)
C	42.4 ± 1.33e	8.715 ± 0.385e	854.04 ± 0.955e	14.14 ± 0.1d	1.55 ± 0.04d
F	38.8 ± 1.04f	7.43 ± 0.602f	805.33 ± 4.91f	12.30 ± 0.1e	1.44 ± 0.02e
T1	74.8 ± 0.929 a	17.9 ± 0.35a	1866.3 ± 1.04a	18.64 ± 0.09a	2.41 ± 0.01a
T2	70.0 ± 0.5b	14.74 ± 1.06b	1510.0 ± 9.45b	17.73 ± 0.13b	2.06 ± 0.05b
T3	63.9 ± 2.65c	12.4 ± 0.896c	1202.3 ± 7.51c	17.67 ± 0.07b	2.02 ± 0.02b
T4	58.4 ± 2.43d	10.6 ± 0.332d	1179.3 ± 16.029d	15.17 ± 0.02c	1.86 ± 0.11c

According to the Duncan multiple range test (*p* ≤ 0.05), columns denoted by a same letter are not significantly different. *C *control untreated and infected, *F *fungicide, *T1* *Trichoderma* growing on medium supplemented with potassium tartrate, *T2* *Trichoderma* growing on medium supplemented with micronutrients, *T3* *Trichoderma* growing on medium supplemented with thiamine, *T4* *Trichoderma* control

### Impact on oxidation indicators

**Fig. 2:**
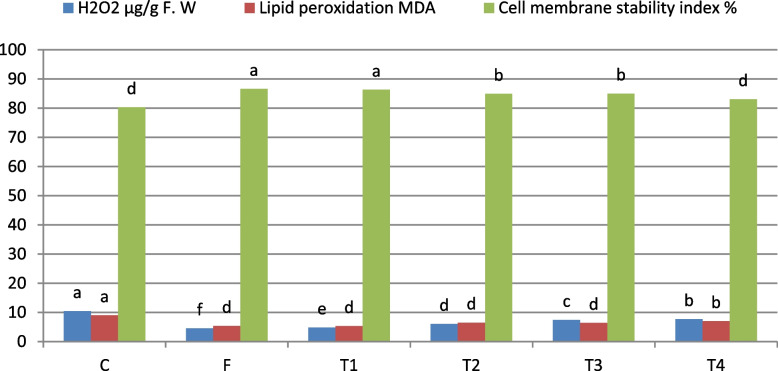
Effect of TSDCIS on H_2_O_2_, lipid peroxidation, cell membrane stability, and ascorbic acid in the leaves of wheat plants (Morocco genotype) infected with *Puccinia* *graminis* f. sp. *tritici* 14 days after infection at the seedling stage under greenhouse conditions

Figure 2 illustrates the effects of various treatments on oxidative stress and membrane stability in wheat plants (Morocco genotype) infected with Pgt 14 days post-infection. The untreated control exhibited the highest hydrogen peroxide (H₂O₂) levels (10.43 µg/g F.W), indicating severe oxidative stress. In contrast, the fungicide and T1 significantly reduced H₂O₂ levels to 4.53 and 4.84 µg/g F.W, respectively. T2 and T3 showed moderate reductions, while T4 had limited efficacy. Lipid peroxidation measured as MDA content, was highest in the control (9.00 µg/g F.W), reflecting substantial membrane damage. Both F and T1 significantly reduced MDA levels to 5.4 and 5.3 µg/g F.W, respectively, while T2 and T3 provided intermediate protection. T4 also reduced MDA levels, its effect was less pronounced.

Cell membrane stability (CMS) was lowest in the control (80.3%), indicating severe membrane disruption. The fungicide and T1 exhibited the highest CMS values (86.6% and 86.3%), with T2 and T3 slightly behind. T4 showed moderate improvement (83.05%) but remained less effective than other treatments. In summary, T1 was comparable to the fungicide in reducing oxidative damage and improving membrane stability, demonstrating its potential as a sustainable alternative for managing stem rust in wheat.

### Impact on photosynthetic pigments content

**Fig. 3:**
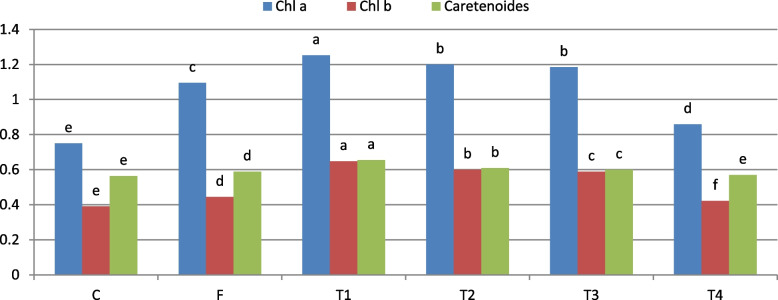
Effect of TSDCIS on photosynthetic pigments content in the leaves of wheat plants (Morocco genotype) infected with *Puccinia* *graminis* f. sp. *tritici *three days after infection at the seedling stage under greenhouse conditions

Figure 3 illustrates the effects of different treatments on photosynthetic pigments levels, including Chlorophyll a, Chlorophyll b, and carotenoids, in wheat leaves infected with Pgt three days post-infection. The untreated control exhibited the lowest pigment concentrations, with Chlorophyll a at 0.751 µg/g, Chlorophyll b at 0.390 µg/g, and carotenoids at 0.564 µg/g. These reduced levels indicate severe impairment of the photosynthetic machinery, and its detrimental impact on plant vitality.

In contrast, T1 treatment resulted in the highest pigment levels, with Chlorophyll a at 1.252 µg/g, Chlorophyll b at 0.648 µg/g, and carotenoids at 0.655 µg/g, demonstrating superior protection and enhanced photosynthetic efficiency. T2 and T3 also improved pigment content, with T2 outperforming T3 slightly. The fungicide treatment showed intermediate effects, with moderate increases in all pigment levels compared to the untreated control, though not as effective as T1. T4 displayed limited efficacy, with only slight improvements over the control.

In summary, T1 was the most effective treatment in mitigating Pgt-induced photosynthetic damage, leading to pigment levels that exceeded those achieved by the fungicide and other Trichoderma-based treatments. This underscores its potential as a sustainable strategy for preserving photosynthetic efficiency and enhancing wheat resilience against Pgt.

### Impact on diseases parameters under field conditions

The data in Table 2 evaluate the effects of TSDCIS treatments on disease severity (DS) and AUDPC for WSR in the Morocco genotype over two growing seasons (2021/2022 and 2022/2023). The results indicate substantial differences in treatment efficacy. The untreated control had the highest DS (96.7% and 98.3%) and AUDPC values (1460.2 and 1492.4) across the two seasons, confirming the Morocco genotype’s extreme susceptibility to WSR. In contrast, the fungicide (F) completely suppressed the disease, reducing DS and AUDPC by 100%, establishing it as the benchmark for effective disease control.

**Table 2:** Effect of TSDCIS on the disease parameters of stem rust Morocco genotype 2021/2022 and 2022/2023 growing seasons

Treatment	Disease severity %			AUDPC		
	First season	Second Season	Efficacy %	First season	Second season	Reduction %
C	96.7 ± 1.0 a	98.3 ± 2.9 a	00.0	1460.2 ± 2.0 a	1492.4 ± 1.2a	00.0
F	00.0 ± 0.0f	00.0 ± 0.0f	100	00.00 ± 0.0f	00.00 ± 0.0f	100
T1	20.0 ± 1.0e	23.3 ± 1.0e	77.7	320.5 ± 1.0e	344.0 ± 1.0e	77.5
T2	23.3 ± 1.0 d	26.7 ± 1.0 d	74.4	370.0 ± 5.0d	390.0 ± 5.0d	74.3
T3	30.0 ± 1.0c	33.3 ± 0.3c	64.5	470.0 ± 5.0.0c	510.0 ± 4.0c	66.8
T4	40.0 ± 0.1b	43.3 ± 1.0b	57.2	616.7 ± 1.0b	666.7 ± 1.0b	56.5

According to the Duncan multiple range test (*p* ≤ 0.05), columns denoted by a same letter are not significantly different. *C *control untreated and infected, *F *fungicide, *T1* *Trichoderma* growing on medium supplemented with potassium tartrate, *T2* *Trichoderma* growing on medium supplemented with micronutrients, *T3* *Trichoderma* growing on medium supplemented with thiamine, *T4* *Trichoderma* control

Among *Trichoderma* treatments, T1 was the most effective biocontrol treatment, reducing DS to 20.0% in the first season and 23.3% in the second season, while decreasing AUDPC values to 320.5 and 344.0, respectively. These reductions correspond to 77.7% DS suppression and 77.5% AUDPC suppression compared to the control. Notably, T1’s efficacy approached that of the fungicide, demonstrating the enhanced biocontrol potential of potassium tartrate-supplemented *Trichoderma*. Other *Trichoderma* treatments also exhibited varying degrees of efficacy. T2 was the second most effective, reducing DS by 74.4% and AUDPC by 74.3%. T3 provided moderate suppression, while T4 had the lowest impact, reducing DS by 57.2% and AUDPC by 56.5%.

### Impact on yield parameters

Table 3 illustrates the significant impact of TSDCIS treatments on wheat grain yield and 1000-kernel weight across two consecutive growing seasons (2021/2022 and 2022/2023). These findings underscore the enhanced performance of *Trichoderma*-based biocontrol treatments compared to the untreated control and the fungicide benchmark. The untreated control exhibited the lowest grain yield (0.987 kg in the first season and 0.983 kg in the second season) and kernel weight (19.9 g and 20.6 g), serving as a baseline for evaluating treatment efficacy.

**Table 3:** Effect of TSDCIS on the yield parameters of Morocco genotype during the 2021/2022 and 2022/2023 growing seasons under field conditions

Treatment	Grain yield (kg)			Weight of 1000 kernel (g)			
	First season	Second Season	Increase %		First season	Second Season	Increase %
C	0.987±0.002^e^	0.983±0.0025^e^	000		19.9±0.002 ^f^	20.6±0.135^e^	00.00
F	2.44±0.005 ^a^	2.48±0.0046^a^	148.7		41.38± 0.005^a^	43.0 ±0.575^a^	107.9
T1	1.9.0±0.005^b^	2.0±0.005^b^	116.2		34.8±0.005 ^b^	35.4±0.041^b^	72.9
T2	1.7±0.0052^c^	1.8±0.0025^c^	98.0		31.5 ±0.00^c^	31.9±0.085^c^	56.2
T3	1.5±0.0151^c^	1.6±0.0072^c^	79.7		29.8± 0.015^d^	30.4±0.036^d^	48.3
T4	1.47±0.00152d	1.55±0.0053^d^	53.2		28.6± 0.0015^e^	28.9±0.050^e^	41.6

According to the Duncan multiple range test (*p* ≤ 0.05), columns denoted by a same letter are not significantly different. *C* control untreated and infected, *F *fungicide, *T1* *Trichoderma *growing on medium supplemented with potassium tartrate, *T2* *Trichoderma* growing on medium supplemented with micronutrients, *T3* *Trichoderma* growing on medium supplemented with thiamine, *T4* *Trichoderma* control

In contrast, the fungicide treatment achieved the highest yield (2.44 kg and 2.48 kg) and kernel weight (41.38 g and 43.0 g), indicating a 148.7% increase in grain yield and a 107.9% increase in kernel weight compared to the control. These results affirm the fungicide's effectiveness as a conventional treatment standard. Among the *Trichoderma*-based treatments, T1 outperformed all others, with grain yield improvements of 116.2% and kernel weight increases of 72.9% over the untreated control. T2 demonstrated notable efficacy, with grain yield improvements of 98% and kernel weight increases of 56.2%. T3 (thiamine supplementation) and T4 showed moderate results, with grain yield and kernel weight increases ranging between 41.6% and 79.7%.

### Electron microscopic observations

**Fig. 4:**
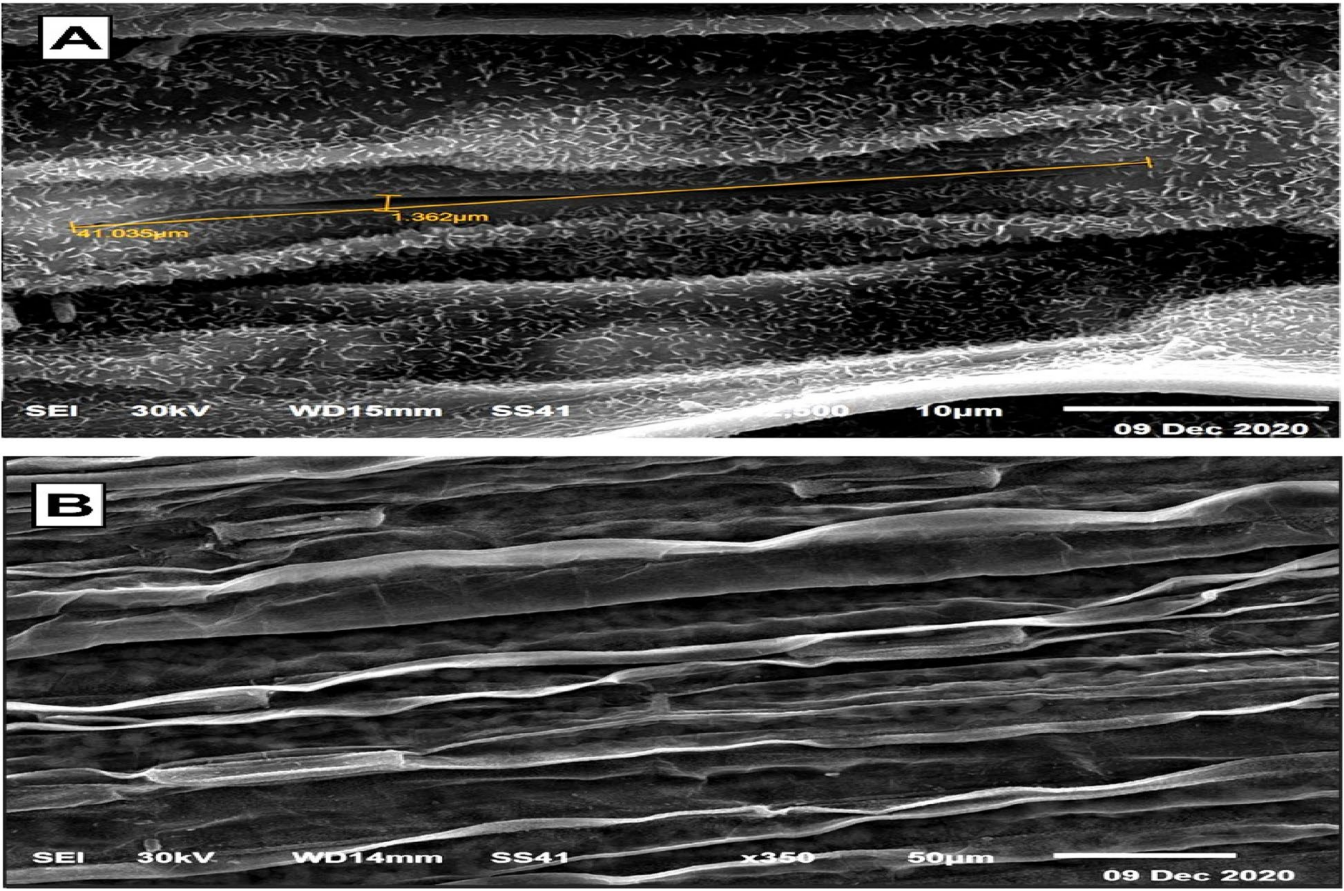
Scanning electron micrographs (SEM) depict stomatal behavior in wheat leaves under control conditions, serving as a baseline for untreated plants. (**A**) Opened stomata with a visible and pronounced stomatal pore area reflect the natural physiological state of the plant, with no intervention to regulate water loss or gas exchange. (**B**) Closed stomata are observed less frequently, indicating minimal stress-induced stomatal closure. These images highlight the susceptibility of untreated wheat leaves to water loss and potential pathogen invasion due to less regulated stomatal activity

**Fig. 5:**
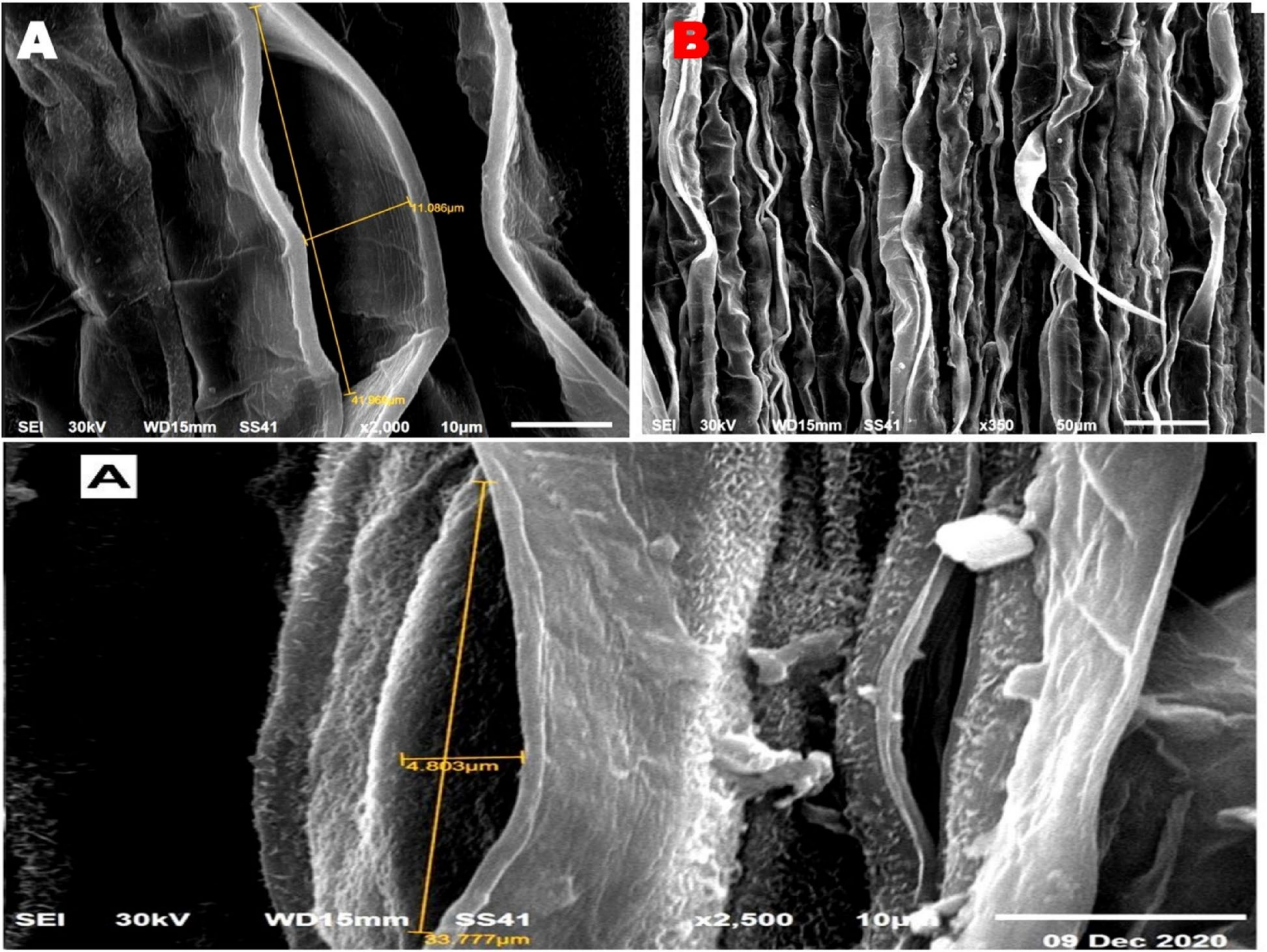
SEM micrographs show the impact of T1 treatment on stomatal dynamics in wheat leaves. (**A**) Opened stomata in T1-treated plants exhibit a significant reduction in stomatal pore area compared to the control, demonstrating the treatment’s ability to modulate water loss and gas exchange under stress conditions. (**B**) Closed stomata are more prevalent in T1-treated plants, reflecting an enhanced physiological response aimed at conserving water and optimizing stress adaptation. These findings underline the role of T1 in improving stomatal regulation to bolster plant resilience

**Fig. 6:**
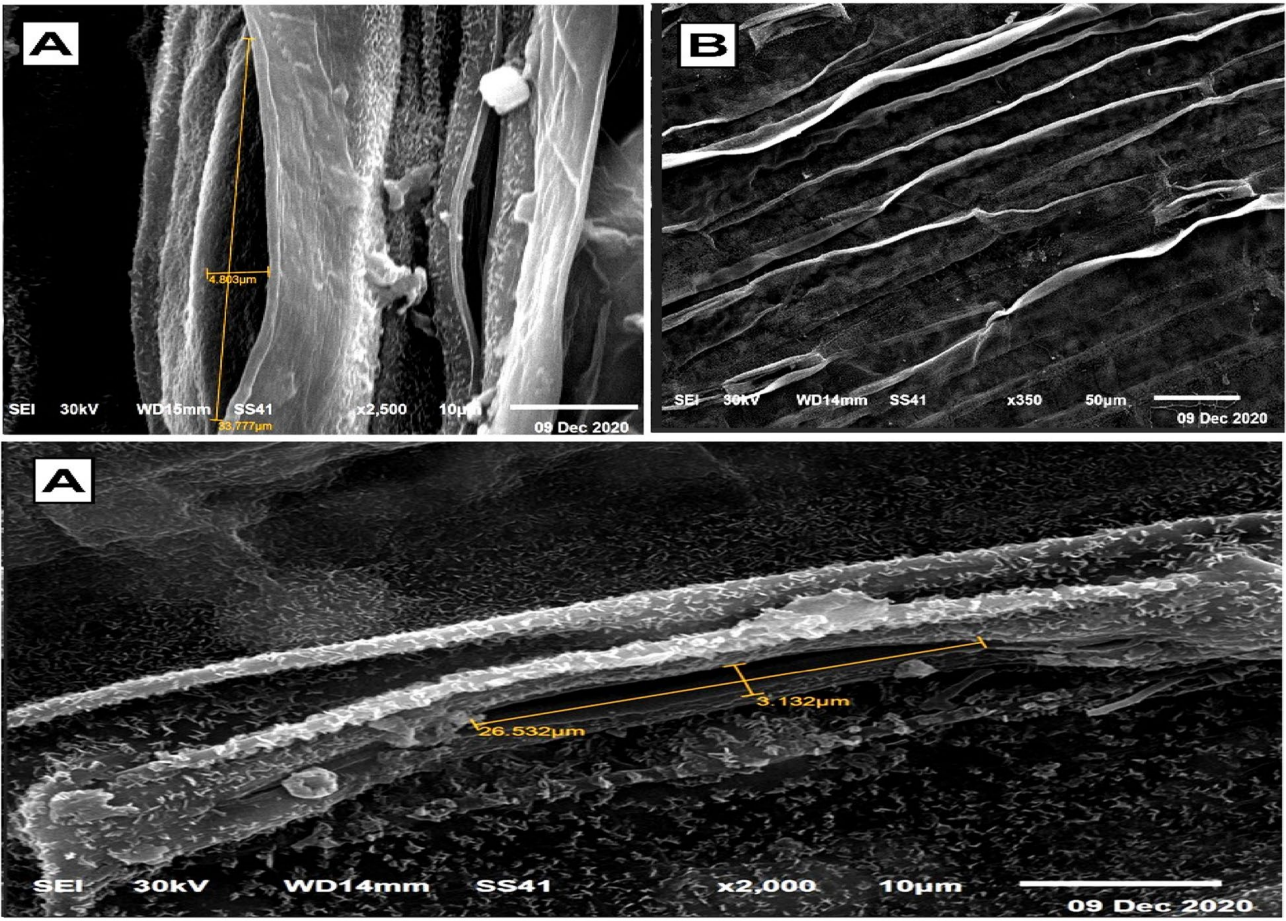
SEM illustrating stomatal behavior in wheat leaves under T1 treatment during the first season. (**A**) opened stomata with reduced stomatal pore area, highlighting the regulated water-loss strategy induced by T1. (**B**) closed stomata, reflecting the plant's enhanced response to stress conditions. These micrographs emphasize the role of T1 in modulating stomatal dynamics to optimize water retention and improve overall plant resilience

**Fig. 7:**
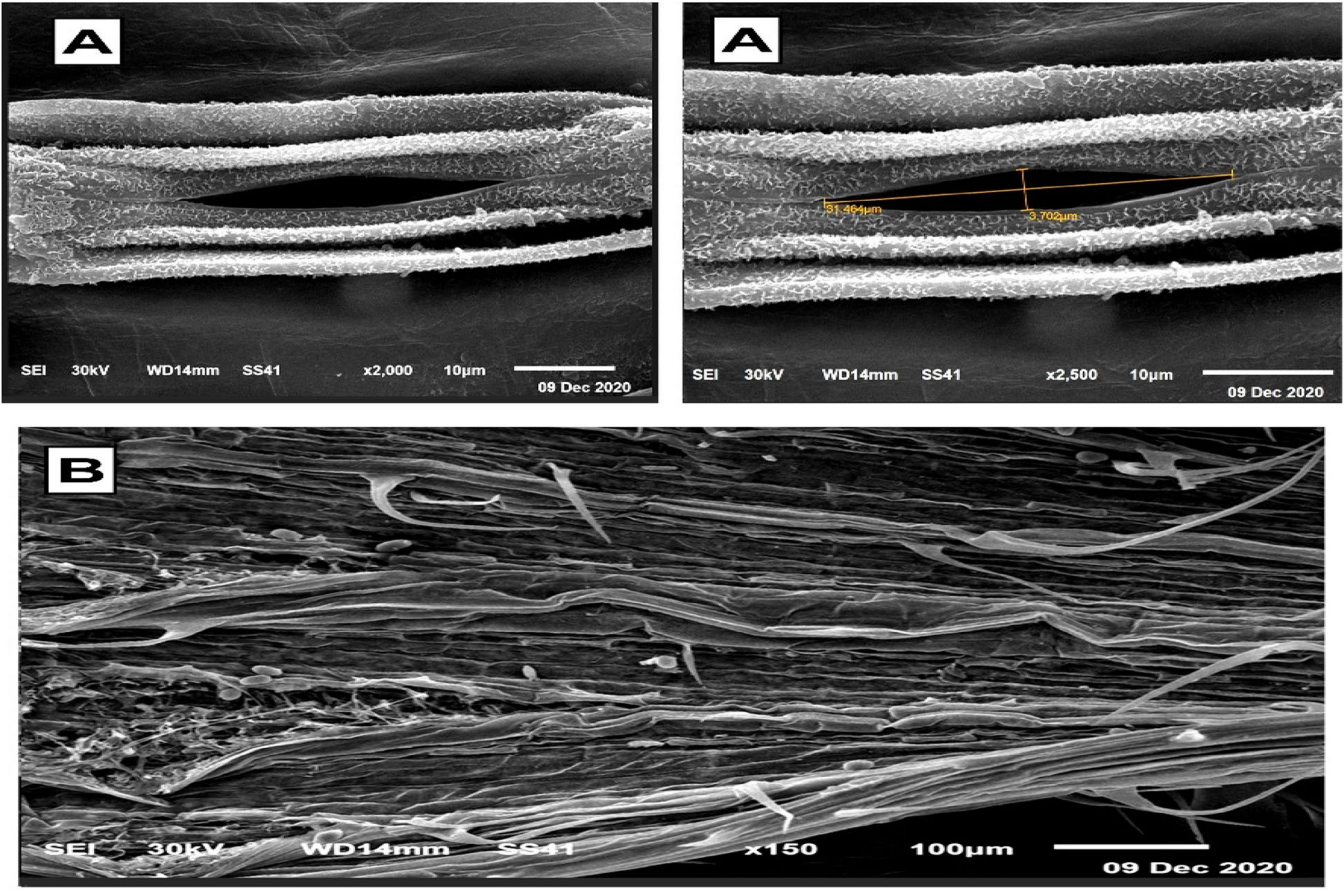
SEM showing stomatal behavior in wheat leaves under T1 treatment during the second season. (**A**) opened stomata with a noticeable reduction in pore area, indicating improved stomatal regulation. (**B**) closed stomata, demonstrating enhanced water-use efficiency and stress adaptation under T1 treatment. These observations highlight the consistency of T1's impact on stomatal dynamics across different growing seasons, contributing to improved plant resilience

The SEM and TEM micrographs, along with the data in Table 4, illustrate the significant impact of T1 treatment on stomatal regulation and cellular ultrastructure in wheat leaves. Figures 4 and 5 reveal that untreated control plants exhibit wide stomatal pores with minimal closure, making them more susceptible to water loss and pathogen invasion. In contrast, Figs. 6 and 7 show that T1-treated plants consistently exhibit reduced stomatal pore areas and a higher frequency of closed stomata, indicating improved water-use efficiency and enhanced stress adaptation across seasons.

**Table 4:** SEM observations on the average number of closed and opened stomata in the leaves of wheat infected with Pgs in response to treatment with T1

Treatment	Stomatal number	
	Opened	Closed
C	169.0 ± 1a	107.0 ± 1b
T1	135.0 ± 1b	115.0 ± 1a

**Fig. 8:**
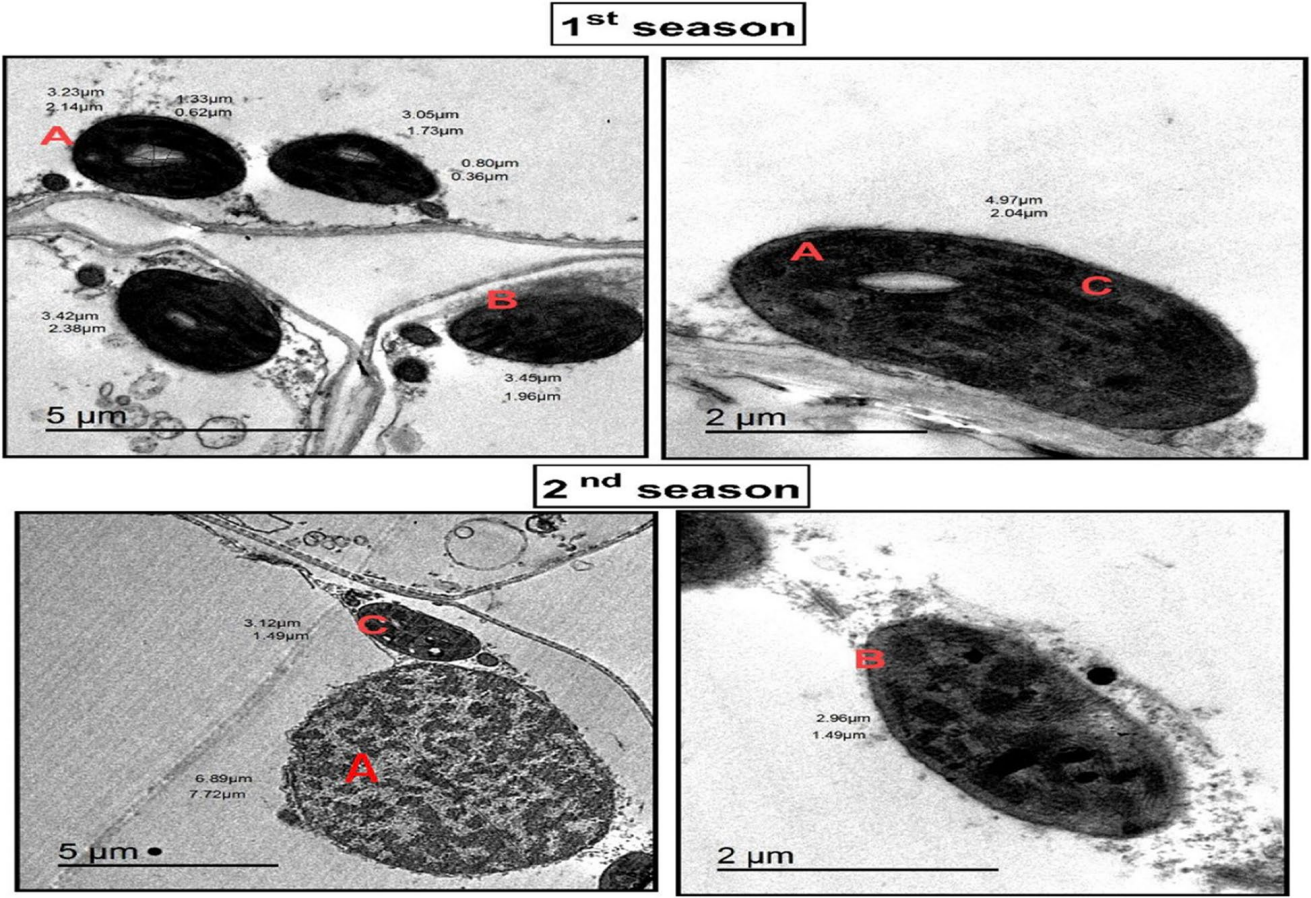
TEM presents micrographs of wheat leaf cells subjected to control treatment during the first and second seasons, revealing pronounced ultrastructural damage and cellular dysfunction. The micrographs illustrate (**A**) complete plasmolysis, where the plasma membrane detaches entirely from the cell wall, indicating severe dehydration and loss of turgor pressure. (**B**) Disruption of cellular components is evident, with vacuoles and cytoplasmic content appearing fragmented and disorganized, reflecting impaired metabolic activities and structural instability. (**C**) abnormal chloroplasts are observed, characterized by distorted shapes, loss of thylakoid membrane integrity, and accumulation of plastoglobules, which suggest disrupted photosynthetic efficiency and impaired energy metabolism. These findings underscore the vulnerability of wheat leaf cells under control conditions, highlighting the extent of damage to cellular and chloroplast structures that compromise overall cell viability and functionality. This damage results from increased r pathogen susceptibility, further emphasizing the need for protective treatments to maintain cellular integrity and improve plant resilience

**Fig. 9:**
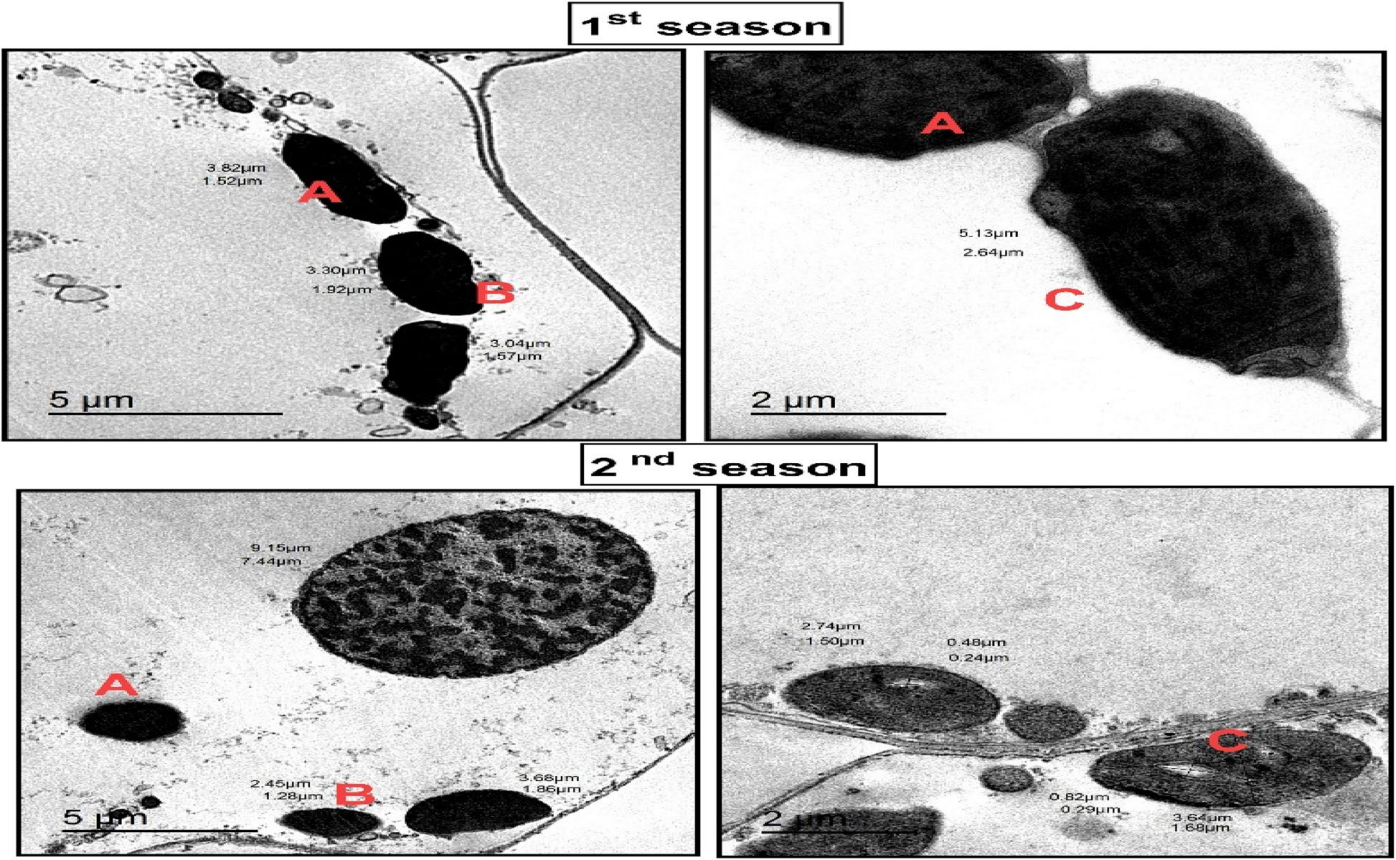
TEM micrographs demonstrating the impact of T1 treatment on the ultrastructure of wheat leaf cells during the first and second growing seasons. In untreated control plants, chloroplasts exhibited severe malformations, including disorganized thylakoid membranes, loss of membrane integrity, and complete plasmolysis, signifying significant cellular dysfunction. In contrast, T1-treated plants showed enhanced structural integrity, with intact thylakoid membranes, reduced plasmolysis, and improved chloroplast morphology. While some abnormalities in chloroplast structure were noted under T1 treatment, the overall preservation of cellular and chloroplast integrity highlights the protective role of T1 in mitigating pathogen-induced injuries, maintaining cellular homeostasis, and potentially enhancing plant resilience and performance

TEM images in Figs. 8 and 9 further highlight the striking differences in cellular ultrastructure. Control plants show severe plasmolysis, fragmented cytoplasmic content, and malformed chloroplasts, leading to compromised photosynthesis and cellular dysfunction. In contrast, T1-treated plants maintain intact cellular membranes, well-structured chloroplasts, and reduced plasmolysis, demonstrating better preservation of cellular integrity and function. The quantitative data in Table 7 reinforce these findings, showing notable improvements in stomatal behavior, cellular stability, and overall plant performance in response to T1 treatment. These results underscore T1’s critical role in enhancing plant resilience under stress conditions, making it a promising strategy for improving wheat tolerance to biotic and abiotic stressors.

## Discussion

The findings demonstrated that, under both greenhouse and field conditions, all tested treatments significantly improved TH’s ability to suppress WSR, stimulate plant growth, and boost yield. Among the treatments, potassium tartrate (T1) elicited a robust defense response, activating biochemical, structural, and molecular mechanisms that collectively enhanced plant resistance and reduced disease severity. These results highlight the effectiveness of integrating TH with targeted chemical additives as a sustainable and eco-friendly strategy for disease management and agricultural productivity improvement.

### Mechanisms of action

Beyond Induced Systemic Resistance (ISR), *Trichoderma* exhibits direct antifungal activity by producing secondary metabolites, including chitinases, β−1,3-glucanases, and proteases, which degrade pathogen cell walls and inhibit fungal growth [24–26]. The supplementation of *Trichoderma* with potassium tartrate (T1) enhances the secretion of these antifungal enzymes, further strengthening its biocontrol efficacy against wheat stem rust. Additionally, volatile organic compounds such as 2,3-butanediol and harzianic acid play a crucial role in pathogen suppression by inhibiting fungal development [3, 27].

The defense response triggered by T1 involved biochemical, structural, and molecular mechanisms that collectively enhanced plant resistance and reduced disease severity. Transcriptomic and proteomic analyses confirmed that *T. harzianum* treatments activated key resistance pathways [26]. Additionally, essential defense-related enzymes—including cellulase, chitinases, 1,3-glucosidases, and ligninase—were upregulated, restricting fungal development [26, 28]. The synthesis of antifungal metabolites, such as pyrones, viridian, koninginins, and gliovirin, was also significantly increased, further contributing to pathogen inhibition [4, 25]. Moreover, *T. harzianum*-treated plants exhibited elevated phenolic compound levels, reinforcing structural defenses and mitigating oxidative stress [26, 29].

The role of salicylic acid (SA) and jasmonic acid (JA) pathways in plant defense was evident in this study, with enhanced activity of antioxidant enzymes, including polyphenol oxidase (PPO) and peroxidase (POD), and increased ascorbic acid levels contributing to ROS scavenging and oxidative stress reduction. This was accompanied by decreased hydrogen peroxide accumulation, lipid peroxidation, and electrolyte leakage, preserving cellular integrity and limiting pathogen-induced damage. Among the tested treatments, T1 exhibited the highest disease suppression efficacy, likely due to its role in enhancing *Trichoderma* metabolic activity and increasing the production of bioactive compounds, including hydrocarbons, esters, and terpenes [7]. Comparatively, T3 (thiamine) enhanced systemic resistance pathways, while T2 (micronutrients) supported Trichoderma growth and bioactivity under varying environmental conditions. These findings align with previous studies [3, 7] (El-Sharkawy et al., 2023; Yousef et al., 2018), which demonstrated that chemical inducers enhanced *T. harzianum*’s antagonistic activity against *Plasmopara viticola* and *Rhizoctonia solani*.

*Trichoderma* plays a key role in plant defense by modulating jasmonic acid (JA) and salicylic acid (SA) pathways. JA enhances resistance against necrotrophic pathogens, while SA is essential for defending against biotrophic pathogens like *P. striiformis f. sp. tritici* [26]. Reactive oxygen species (ROS), including H₂O₂, O₂⁻, and hydroxyl radicals, act as secondary messengers, triggering MAPK pathways and inducing key defense enzymes such as PAL and PR proteins [30]. NADPH oxidase (Nox) is central to ROS production during plant-*Trichoderma* interactions, with NoxR and Nox1 influencing both defense responses and plant growth [31]. While enhanced immunity strengthens pathogen resistance, excessive activation—especially of JA signaling—can reduce plant growth due to energy trade-offs [32, 33]. However, *Trichoderma* maintains a balance between immune activation and growth promotion, ensuring optimal resource allocation.

T1-treated plants showed significant improvements in growth and yield, including increased spike weight, grain weight per spike, and 1000-kernel weight, attributed to Trichoderma's ability to regulate plant hormones, enhance nutrient uptake, and improve photosynthetic efficiency [3]. *Trichoderma* colonizes plant roots, triggering systemic defense responses and optimizing phytohormonal balance, particularly through the modulation of ethylene, salicylic acid, jasmonic acid, abscisic acid, indole acetic acid, and gibberellins [26, 30, 32]. The secretion of 1-aminocyclopropane-1-carboxylate deaminase reduces ET levels, enhancing root elongation and lateral root formation through DELLA protein degradation [32]. Experimental studies confirm *Trichoderma*’s role in boosting plant biomass and root development in various crops [26, 34]. These findings highlight *Trichoderma*’s potential as a sustainable biostimulant, improving plant resilience against biotic and abiotic stresses while enhancing agricultural productivity [35].

Alongside pathogen suppression, *Trichoderma* enhances plant growth by promoting nitrogen and phosphorus acquisition, increasing root surface area, and stimulating auxin production, which supports lateral root formation [32, 33]. Moreover, *Trichoderma* improves iron and zinc solubilization, ensuring optimal micronutrient availability under stress conditions [31].

Supplementing TH with chemical inducers, particularly potassium tartrate significantly enhances photosynthetic efficiency and plant resilience. Studies by El-Sharkawy et al. (3,6) demonstrated that this combination boosts chlorophyll a, chlorophyll b, and carotenoid levels in grapevines and wheat, reinforcing the role of these pigments in energy conversion and yield optimization. This study further highlights the detrimental impact of Pgt infection on wheat, causing pigment reduction, impaired CO₂ diffusion, and chloroplast malformation. However, T1 treatment restored chloroplast integrity, increased grana density, and improved overall photosynthetic performance. Beyond pathogen suppression, T1 enhances photosynthetic machinery by stabilizing chloroplast structures and maintaining pigment levels, aligning with findings from El-Sharkawy et al. (3). These results position potassium tartrate as a promising biostimulant, warranting further research into its role in optimizing photosystem function and carbon assimilation under stress conditions.

T1 treatment significantly enhanced wheat defense mechanisms by regulating stomatal dynamics and reinforcing leaf structural integrity. A key observation was the reduction in stomatal aperture and pore area, leading to an increased number of closed stomata, which restricted pathogen entry. This aligns with studies showing that *Trichoderma* releases volatile organic compounds (VOCs) like 2,3-butanediol, which induce stomatal closure and strengthen plant immunity [3, 36]. Beyond stomatal regulation, T1 treatment improved leaf structural defenses, including thickened mesophyll cell walls and enhanced cellular organization, creating a robust barrier against pathogen invasion. Additionally, chloroplast ultrastructure was optimized, with increased grana density and restored integrity, ensuring higher photosynthetic efficiency under stress. These findings underscore T1’s dual role in pathogen resistance and physiological resilience, positioning it as a powerful tool for sustainable crop protection.

### Field implications and scalability

The estimated cost of T1 treatment is 250 EGP per feddan, which equates to 595 EGP per hectare (≈ 11.9 USD per hectare, based on an exchange rate of 1 USD = 50 EGP). In contrast, the cost of Crown fungicide, a commonly used chemical fungicide for wheat rust management, is 380 EGP per feddan, translating to 904 EGP per hectare (≈ 18.08 USD per hectare). This analysis highlights that T1 treatment is approximately 34% more cost-effective than Crown fungicide. Additionally, T1 offers long-term benefits such as soil health improvement, enhanced plant growth, and reduced environmental impact, making it a sustainable and economically viable alternative for wheat rust management.

For large-scale application, *T. harzianum* can be mass-produced using solid-state or submerged fermentation, ensuring high spore viability and cost efficiency. Optimized formulations in dry powder, granule, or liquid suspension forms improve storage stability and ease of use. Application methods such as seed coating, foliar spraying, and soil drenching offer flexibility, while precision agriculture techniques like drip irrigation and controlled foliar spraying enhance efficiency across extensive farms.

Unlike chemical fungicides, *T. harzianum* is a biocontrol agent with minimal environmental risk, promoting soil microbiome diversity and organic matter decomposition. T1 supplementation enhances these benefits without introducing chemical residues or contributing to groundwater contamination. Additionally, *T. harzianum* induces systemic resistance, reducing the need for repeated fungicide applications and lowering the overall chemical load in agricultural ecosystems. Future research should assess long-term ecological impacts through multi-season field trials across diverse agroecosystems.

Integrating T1 into sustainable disease management programs can enhance disease suppression and crop resilience. Combining T1 with resistant wheat cultivars creates a multi-layered defense strategy, reducing fungicide dependency and pathogen resistance risks. Practices such as crop rotation and intercropping foster a healthier soil microbiome, supporting *T. harzianum* colonization and persistence. Additionally, organic soil amendments, such as biochar and compost, further stimulate microbial activity, reinforcing plant defense mechanisms. T1 also aligns with Integrated Pest Management (IPM) principles, promoting eco-friendly and economically viable disease control. By integrating biological control, genetic resistance, and sustainable agronomic practices, T1 has the potential to revolutionize wheat stem rust management, ensuring higher yields and long-term agricultural sustainability.

### Explore future research directions

While our current study provides strong evidence from both greenhouse experiments and small-scale field trials, large-scale multi-season studies are essential to assess the stability, consistency, and scalability of T1 treatment under varying climatic and soil conditions.

Future field trials will focus on:

- ➢ Evaluating efficacy across different agroecological zones, considering variations in temperature, humidity, and soil properties that may influence *T. harzianum* survival and performance.
- ➢ Assessing long-term sustainability by monitoring pathogen pressure, crop yield, and soil microbiome shifts over multiple growing seasons.
- ➢ Comparing T1 treatment with conventional disease management strategies, including chemical fungicides and IPM approaches, to provide comprehensive cost–benefit analyses.
- ➢ Refining large-scale application techniques, such as precision foliar spraying and seed coating, to ensure efficient and uniform field deployment.

These field studies will provide critical insights into the practical implementation, economic feasibility, and environmental impact of T1 supplementation, paving the way for its adoption as a scalable and sustainable disease management strategy.

While our current study provides strong physiological and biochemical evidence, further research using gene expression analysis, transcriptomics, proteomics, and metabolomics is essential to gain deeper insights into the molecular basis of T1’s biocontrol and plant growth-promoting effects.

Future molecular investigations will focus on:

- ➢ Gene Expression Analysis: Utilizing qRT-PCR and RNA-seq to identify key defense-related genes upregulated in response to T1 treatment, particularly those involved in induced systemic resistance (ISR), phytohormone signaling (JA, SA, and ET pathways), and secondary metabolite biosynthesis.
- ➢ Proteomics and Metabolomics: Conducting mass spectrometry-based proteomic and metabolomic profiling to characterize the bioactive compounds produced by *T. harzianum* in the presence of T1, including antifungal metabolites (e.g., chitinases, β−1,3-glucanases) and plant defense elicitors.
- ➢ Epigenetic Modifications: Exploring whether T1 supplementation influences DNA methylation or histone modifications, leading to long-term priming of plant defense responses against wheat stem rust.
- ➢ Fungal-Plant Interaction Studies: Investigating the molecular dialogue between *T. harzianum* and wheat plants using dual RNA-seq, which will provide insights into how T1 enhances fungal colonization, root signaling, and systemic resistance activation.

By integrating these molecular approaches, we aim to uncover novel regulatory networks and biochemical pathways influenced by T1, thereby strengthening the scientific basis for its biocontrol efficacy and plant growth-promoting potential. These findings will be crucial in optimizing T1 formulations and developing next-generation biofungicides with improved field performance and environmental safety.

## Conclusion

This study demonstrates that *T. harzianum* supplemented with potassium tartrate (T1) is a promising and sustainable strategy for managing wheat stem rust. By enhancing both structural and biochemical plant defenses, T1 effectively reduces disease severity and improves crop productivity. Integrating T1 into IPM programs can minimize reliance on chemical fungicides while maintaining effective disease control.

To ensure its practical applicability, large-scale field trials are needed to validate its efficacy across diverse agroecosystems. Further research should also assess its long-term ecological impact and explore its potential in other economically important crops. Additionally, investigating complementary chemical inducers and the molecular mechanisms underlying T1-induced resistance could optimize its biocontrol efficiency. Raising farmer awareness will be crucial for its widespread adoption. This study provides a foundation for developing scalable, eco-friendly, and scientifically validated wheat disease management strategies.

## Data Availability

All authors declare that the obtained data in this study will be available upon reasonable request.

## Abbreviations

WSR: Wheat Stem Rust
TH: *Trichoderma harzianum*
PDB: Potato dextrose broth
TSDCIS: *Trichoderma harzianum* Grown on PDB medium supplemented with chemical inducers
CMS: Cell membrane stability
SEM: Scanning Electron Microscopy
TEM: Transmission Electron Microscopy
ISR: Induced Systemic Resistance
PPO: Polyphenol Oxidase
POD: Peroxidase
DS: Disease severity
IPM: Integrated Pest Management
SA: Salicylic acid
JA: Jasmonic acid
CRBD: Completely randomized block design
